# Long-term changes in bone mineral density and associated risk factors in individuals with spinal cord injury: A retrospective study

**DOI:** 10.1097/MD.0000000000039790

**Published:** 2024-09-27

**Authors:** Chaeun Mun, Keunyoung Sho, Onyoo Kim

**Affiliations:** a Department of Physical Medicine and Rehabilitation, National Rehabilitation Center, Seoul, Republic of Korea.

**Keywords:** absorptiometry, bone mineral density, osteoporosis, risk factors, spinal cord injury

## Abstract

Individuals with spinal cord injury (SCI) experience a notable decrease in bone mass below the level of injury. While studies have primarily focused on the acute phase with a small cohort, this study aimed to provide comprehensive insights into bone loss patterns over time. A total of 427 individuals with SCI who underwent dual-energy X-ray absorptiometry (DXA) testing at the Korea National Rehabilitation Center (2010–2021) were included and analyzed by categorizing the DXA results into 1-year intervals based on postinjury duration. Demographic characteristics (age, sex, body mass index, and alcohol/smoking history) and SCI-related factors (etiology, severity, extent of injury, motor score, and Korean Spinal Cord Independence Measure 3rd edition) were collected and analyzed. Linear mixed models and Bonferroni post hoc tests were performed to assess temporal changes in bone mass and linear regression analysis to assess the associations between possible risk factors and bone loss. DXA results revealed that substantial annual bone loss occurred in the total hip site up to 3 years postinjury and in the femoral neck site up to 2 years postinjury. Old age, women, and low body mass index were significant risk factors for bone loss in the SCI population. Additionally, during the chronic phase, lower Korean Spinal Cord Independence Measure 3rd edition scores were associated with low bone mass. Significant annual bone loss in the hip region persists for up to 3 years postinjury in individuals with SCI. While prioritizing the risk factors for osteoporosis commonly used in the general population, applying the SCIM score in the chronic phase may provide additional information on bone loss risk.

## 1. Introduction

After spinal cord injury (SCI), the sudden onset of paralysis leads to an immediate alteration in bone metabolism, characterized by an imbalance between bone formation and resorption processes.^[1–4]^ This rapid bone loss primarily occurs over 12 months, with bone mineral density (BMD) losses of up to 20 to 40%.^[5,6]^ Under dual-energy X-ray absorptiometry (DXA)-measured criteria, 82% of individuals with SCI were diagnosed with osteoporosis or osteopenia.^[7]^ Bone loss in individuals with SCI can lead to devastating consequences, such as severe osteoporosis, fragility fractures, pressure injuries, and prolonged hospitalization.^[8,9]^ Preventing osteoporosis is feasible if it is detected and treated early. Therefore, addressing bone loss following SCI is of paramount clinical importance for reducing the burden of SCI-related complications.

Changes in bone mass from the acute to the chronic phase of SCI remain poorly understood. In addition, most studies were confined to a smaller number of subjects (within the tens).^[10–16]^ Some studies have suggested that bone loss after SCI stabilizes and plateaus 1 to 2 years after injury,^[12,13]^ whereas recent studies have reported ongoing bone loss.^[14–16]^ In a study of 100 men with motor-complete paraplegia, the BMD of the distal tibial diaphysis continued to decrease for over 10 years after injury.^[8]^

Bone loss after injury in the SCI population may be influenced by both demographic and SCI-related factors, which can be strategically targeted to mitigate the risk of osteoporosis and related complications. However, previous studies have produced heterogenous results. Some studies have suggested that time from injury is more important than age or sex,^[17,18]^ whereas other studies have linked old age with lower BMD in post-SCI patients.^[19,20]^ Factors related with osteoporotic fractures have been proposed in a study using data from the Veteran Affairs Spinal Cord Dysfunction Registry, such as White race, traumatic etiology, paraplegia, complete extent, longer duration of SCI, and use of anticonvulsant and opioids.

This study aimed to clarify long-term bone loss from the acute (within 12 months) to the chronic (over 12 months) phase in hundreds of individuals with SCI and to identify both demographic and SCI-related factors associated with bone loss during these phases. This information could help develop management strategies based on the identified risk factors to reduce the incidence and progression of osteoporosis in individuals with SCI.

## 2. Methods

### 2.1. Study group

We reviewed the medical records of 1940 individuals diagnosed with SCI who underwent DXA at the Korea National Rehabilitation Center between 2010 and 2021. Among the 440 participants who underwent DXA testing at least twice at this center, individuals aged below 19 (n = 3) were excluded. One individual diagnosed with osteoporosis before the injury and one whose testing interval was only 6 days were excluded. Participants lacking DXA data or personal information (n = 8) were also excluded. Consequently, 427 participants were finally included in the analysis. They were devoid of any comorbidity that could potentially affect bone health, such as rheumatoid arthritis, hyperparathyroidism, hypogonadism, Cushing syndrome, metastatic tumors, and Parkinson disease. Ethics approval was obtained from the Korean National Rehabilitation Center Institutional Review Board (IRB no. NRC-2022-02-010). Informed consent was obtained from all participants involved in the study.

### 2.2. DXA

BMD was measured using DXA (Lunar, GE Healthcare, Chicago, IL, from January 1, 2010, to January 20, 2019; Hologic Inc., Marlborough, MA, from January 21, 2019, to December 31, 2021) at the lumbar spine, femoral neck, and total hip. Standardization formulas were used to convert the BMD values obtained from the 2 different DXA machines. We used conversion equations as follow: for lumbar spine, H = 0.918 × G − 0.038; for femoral neck, H = 0.8638 × G − 0.039; for total hip, H = 0.971 × G − 0.037 (H = BMD for Hologic Inc., G = BMD for GE Lunar machine).^[21]^ The BMD value was reported in g/cm^2^, and the T-score, which indicates the standard deviation unit for a young, healthy population, was calculated based on the third National Health and Nutrition Examination Survey.^[22,23]^ All DXA tests were performed by 2 technicians using the same ISCD guidelines,^[24]^ and the analyses of DXA results were conducted by the same physician. Values measured at the lumbar spine were excluded from the analysis in cases where potential pitfalls and artifacts (such as degenerative changes or spinal operations) were identified.

The DXA results were categorized based on postinjury duration. The DXA scans conducted within the first year after injury were grouped as “<1 year.” Those conducted between 1 and 2 years after injury were included in the “1 to 2 years” group; those between 2 and 3 years, in the “2 to 3 years” group; those between 3 and 4 years, in the “3 to 4 years” group; those between 4 and 5 years, in the “4 to 5 years” group; and finally, those more than 5 years later, in the “over 5 years” group. This study analyzed the temporal changes in BMD and T-scores between these groups.

### 2.3. Variables

Demographic and clinical data of the participants were obtained from medical records, including age, sex, body mass index (BMI, kg/m^2^), alcohol consumption, smoking history, and SCI-related factors. Smoking history was categorized as current smoker or nonsmoker. Heavy drinkers were defined as individuals who either consumed 4 or more drinks on 1 occasion for women and 5 or more drinks for men, or who consumed 8 or more drinks of alcohol per week for women and 15 or more drinks per week for men.^[25,26]^ The etiology of the injury (traumatic or nontraumatic) was investigated through interviews, which were cross-checked with imaging studies and previous medical records. The American Spinal Injury Association (ASIA) impairment scale and ASIA motor scores were evaluated through neurological examination by an experienced physician using the International Standards for Neurological Classification of Spinal Cord Injury.^[27]^ Complete injuries were categorized as American Spinal Injury Association Impairment Scale (AIS) A, and incomplete injuries were categorized as AIS-B, AIS-C, and AIS-D. The sum of the manual muscle testing scores of the bilateral upper limb key muscles was represented as the upper extremity motor score (UEMS), and the sum of the manual muscle testing scores of the bilateral lower limb key muscles was represented as the lower extremity motor score (LEMS). The Korean Spinal Cord Independence Measure III (K-SCIM-III) score was evaluated by a single experienced occupational therapist.^[28,29]^ Demographic characteristics (age, sex, BMI, drinking history, and smoking history) and SCI-related factors (injury etiology, extent of injury, AIS completeness, ASIA motor score, and K-SCIM-III score) were analyzed to identify risk factors for lower BMD following SCI.

### 2.4. Statistical analysis

Temporal changes in BMD and T-scores were analyzed using a linear mixed model (LMM) in the lme4 package. Significant differences in BMD between the 6 time groups were estimated using the estimated marginal mean from the LMM, and univariate and Bonferroni post hoc tests were performed to identify the specific time points at which significant differences appeared. Changes in BMD and T-scores over time at each tested site were quantified and graphically presented. Multiple linear regression analysis was performed to explore possible risk factors for changes in bone mass. Before this step, correlation analyses were performed to identify variables associated with changes in bone mass. Pearson correlation was used for relationships between 2 continuous variables, and point-biserial correlation to examine the relationship between continuous and binary variables. The collected data were analyzed using SPSS 27.0 (IBM Corp., Armonk, NY) and R 4.2.2 (https://www.r-project.org/). Statistical significance was set at a *P* value of <.05.

## 3. Results

### 3.1. Baseline characteristics

Demographic characteristics and clinical features of the participants are presented in Table 1. The majority of participants were men (73.5%), and the predominant etiology of injury was trauma (74.1%). Nontraumatic injuries included nonmetastatic tumor, myelitis, infection, degenerative spondylosis or stenosis, arteriovenous malformation, and postoperative vascular event or unknown causes. Tetraplegia and paraplegia were equally distributed. Incomplete injuries occurred in more than half of the participants (56.7%). The mean UEMS, LEMS, and K-SCIM-III scores are presented in Table 1.

**Table 1:** Demographic characteristics and clinical features.

Characteristics	N = 427	
	M (SD)	N (%)
Age (years)	44 (15.46)	
Sex		
Men		314 (73.5)
Women		113 (26.5)
BMI (kg/m^2^)	22.45 (3.32)	
Alcohol consumption		
Heavy drinker		84 (19.7)
None		343 (80.3)
Smoking history		
Current smoker		105 (24.6)
Nonsmoker		322 (75.4)
Etiology of injury		
Traumatic		306 (71.7)
Nontraumatic		121 (28.3)
Extent of injury		
Tetraplegia		226 (52.9)
Paraplegia		201 (47.1)
AIS		
A		177 (43.3)
B		76 (18.6)
C		67 (16.4)
D		89 (21.8)
ASIA motor score		
UEMS	36.40 (15.79)	
LEMS	10.72 (15.05)	
K-SCIM-III	35.75 (22.41)	

AIS = American Spinal Injury Association Impairment Scale, ASIA = American Spinal Injury Association, BMI = body mass index, K-SCIM-III = Korean Spinal Cord Independence Measure III, LEMS = lower extremity motor score, UEMS = upper extremity motor score.

### 3.2. Temporal changes in BMD

**Figure 1.**
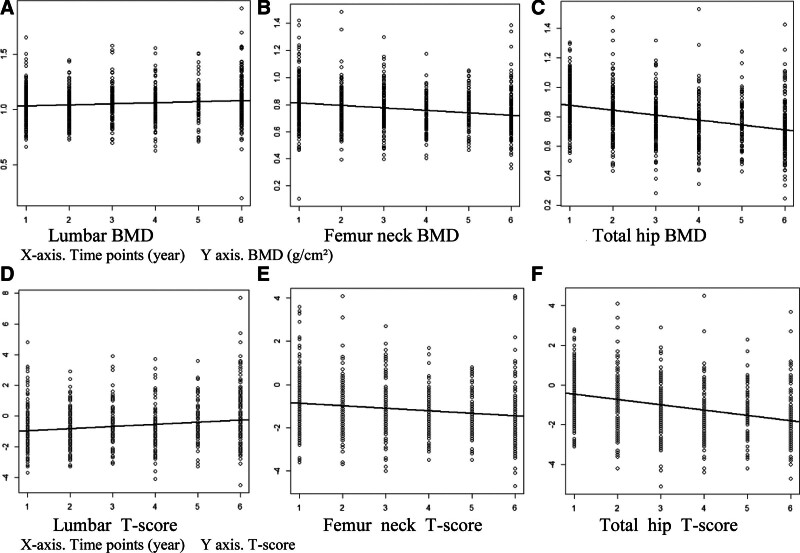
In the lumbar site, both BMD and T-scores showed slight increase over time and, in contrast, in the femur neck and total hip sites, both BMD and T-scores showed negative time effect estimate, indicating significant decrease over time.

LMM indicated significant time-related changes in BMD and T-scores in the lumbar spine, femoral neck, and total hip areas. In the lumbar spine, BMD (*P* = .001) and T-scores (*P* < .001) demonstrated a slight increase over time. In contrast, in the femoral neck, BMD (*P* < .001) and T-scores (*P* < .001) showed a decrease over time. For the total hip, BMD (*P* < .001) and T-scores (*P* < .001) demonstrated a substantial negative time effect estimate, indicating a significant decrease over time (Table 2; Fig. 1).

**Table 2:** Longitudinal changes in bone mineral density and T-scores.

Category	Estimator	Estimate (SE)	T	*P*	95% confidence interval	
					Lower	Upper
Lumbar spine						
BMD (g/cm^2^)	Intercept	1.024 (0.012)	87.651	**<.001**	1.001	1.047
	Time	0.010 (0.003)	3.268	**.001**	0.004	0.015
T-score	Intercept	−1.116 (0.102)	−10.981	**<.001**	−1.316	−0.917
	Time	0.164 (0.026)	6.386	**<.001**	0.114	0.215
Femoral neck						
BMD (g/cm^2^)	Intercept	0.834 (0.010)	81.647	**<.001**	0.814	0.855
	Time	−0.018 (0.003)	−6.814	**<.001**	−0.024	−0.013
T-score	Intercept	−0.744 (0.078)	−9.548	**<.001**	−0.897	−0.591
	Time	−0.016 (0.021)	−5.153	**<.001**	−0.146	−0.065
Total hip						
BMD (g/cm^2^)	Intercept	0.918 (0.011)	82.970	**<.001**	0.896	0.940
	Time	−0.034 (0.003)	−12.449	**<.001**	−0.040	−0.029
T-score	Intercept	−0.190 (0.084)	−2.274	**.023**	−0.354	−0.026
	Time	−0.258 (0.022)	−11.823	**<.001**	−0.301	−0.215

Bold value indicates statistical significance with a *P* value < .05.

BMD = bone mineral density.

### 3.3. Comparison of BMD and T-scores between time groups

DXA results were categorized based on the postinjury duration, as follows: “<1 year,” “1 to 2 years,” “2 to 3 years,” “3 to 4 years,” “4 to 5 years,” and “> 5 years.” Significant differences were observed among these groups in BMD and T-scores for all tested areas: the lumbar spine (*P* = .004 for BMD, *P* < .001 for T-score), femoral neck (*P* < .001 for BMD, *P* < .001 for T-score), and total hip (*P* < .001 for BMD, *P* < .001 for T-score).

Both BMD and T-scores at the lumbar spine exhibited an increasing trend compared with the “<1 year” group to the “>5 years” group (*P* = .014, *P* < .001, respectively). In contrast, both BMD and T-scores at the femoral neck and total hip area decreased. Specifically, BMD at the femoral neck showed a significant decrease over time when comparing the “<1 year” group to the other groups (*P* = .005, *P* < .001, *P* < .001, *P* = .006, *P* < .001, respectively). The T-score at the femoral neck also showed a significant decrease when comparing the “<1 year” group to the other groups (*P* = .016, *P* < .001, *P* < .001, *P* = .011, *P* < .001, respectively). Furthermore, the BMD of the total hip showed a significant decrease when comparing the “<1 year” group to the other groups (*P* = <.001, respectively), and when comparing the “1 to 2 years” group to the other groups, except for the “4 to 5 years” group (*P* = <.001, *P* = .010, *P* = .007, *P* = <.001, respectively). The T-scores for the total hip significantly decreased when comparing the “<1 year” group to the other groups (*P* = <.001, respectively), and when comparing the “1 to 2 years” group to the other groups, except for the “4 to 5 years” group (*P* = <.001, *P* = .012, *P* = .004, *P* = <.001) (Table 3).

**Table 3:** Comparison of bone mineral density and T-scores between groups categorized by postinjury duration.

Time point	Lumbar spine					Femoral neck						Total hip					
	BMD (g/cm^2^)		T-score			BMD (g/cm^2^)			T-score			BMD (g/cm^2^)				T-score	
	N	M	N	M		N	M		N		M	N		M		N	M
<1 year^a^	208	1.042	207	−0.851		253	0.825		252		−0.772	253		0.905		252	−0.279
1–2 years^b^	160	1.030	160	−0.884		178	0.786		178		−1.034	178		0.811		178	−0.993
2–3 years^c^	131	1.045	131	−0.761		151	0.763		151		−1.221	151		0.775		151	−1.255
3–4 years^d^	107	1.046	107	−0.688		112	0.746		111		−1.331	112		0.767		112	−1.331
4–5 years^e^	70	1.068	70	−0.483		78	0.767		78		−1.185	78		0.769		78	−1.296
Over 5 years^f^	150	1.091	150	0.056		163	0.730		163		−1.280	163		0.735		126	−1.520
F (*P*)	3.556 (**.004**)		12.304 (**<.001**)			11.618 (**<.001**)			7.993 (**<.001**)			47.126 (**<.001**)				44.100 (**<.001**)	

	a–b				b–c					e–f					a–f		
	Mean diff (SE)		*P*		Mean diff (SE)			*P*		Mean diff (SE)			*P*		Mean diff (SE)		*P*
Lumbar spine																	
BMD (g/cm^2^)	0.011 (0.011)		1.000		−0.015 (0.012)			1.000		−0.023 (0.014)			1.000		−0.050 (0.015)		**.014**
T-score	0.033 (0.095)		1.000		−0.123 (0.107)			1.000		−0.539 (0.125)			**<.001**		−0.907 (0.131)		**<.001**
Femur neck																	
BMD (g/cm^2^)	0.039 (0.011)		**.005**		0.023 (0.012)			.910		0.037 (0.015)			.198		0.095 (0.014)		**<.001**
T-score	0.262 (0.080)		**.016**		0.186 (0.090)			.596		0.095 (0.110)			1.000		0.507 (0.106)		**<.001**
Total hip																	
BMD (g/cm^2^)	0.095 (0.009)		**<.001**		0.035 (0.010)			**.010**		0.034 (0.013)			.092		0.171 (0.014)		**<.001**
T-score	0.714 (0.069)		**<.001**		0.262 (0.078)			**.012**		0.224 (0.096)			.296		1.241 (0.019)		**<.001**

The values of the lumbar spine, femoral neck, and total hip are from variance analysis.

The adjustments for multiple comparisons are made using the Bonferroni post hoc test.

Bold indicates statistical significance with a *P* value < .05.

BMD = bone mineral density.

### 3.4. Risk factors associated with BMD changes

To identify the factors associated with changes in BMD, we divided the participants into 2 groups: those who underwent initial DXA scans within the first year postinjury (acute phase) and those who underwent the scans after the first year postinjury (chronic phase). In the acute phase group, age (*P* < .001), BMI (*P* = .001), sex (*P* < .001), and injury etiology (*P* = .012) were significantly correlated with BMD changes. In contrast, in the chronic phase group, age (*P* = .002), BMI (*P* = .011), sex (*P* = .009), and K-SCIM-III score (*P* = .026) were significantly correlated with BMD.

To further elucidate their statistical relationship, we reassessed these factors using a linear regression analysis. We observed a significant negative correlation between age and BMD in both acute phase group (β = −0.21, *P* = .001) and chronic phase group (β = −3.39, *P* = .001). BMI showed a slight positive correlation with BMD in the acute phase (β = 0.22, *P* < .001) and chronic phase (β = 3.12, *P* = .002) groups. Sex, coded as 1 for men and 0 for women, demonstrated a significant positive correlation with BMD in both groups (β = 0.19, *P* = .002 for <1-year, and β = 2.35, *P* = .020 for over 1-year). The K-SCIM-III score was positively correlated with BMD in the chronic phase group (β = 2.53, *P* = .012). Unlike in the initial assessment, the etiology of SCI (traumatic = 1) did not show a significant relationship with BMD (β = 0.11, *P* = .087).

The ASIA motor scores (UEMS and LEMS) were not correlated with BMD in either group. Similarly, alcohol consumption, smoking history, injury etiology, extent of injury, and completeness were not significantly correlated with BMD (Table 4).

**Table 4:** Comparison of risk factors contributing to decreased bone mineral density.

Acute phase					Chronic phase				
Variables	Correlation		*P* value		Variables	Correlation		*P* value	
Age (years)	−0.243		<.001*		Age (years)	−0.245		.002*	
BMI (kg/m^2^)	−0.201		.001*		BMI (kg/m^2^)	0.199		.011*	
ASIA motor score					ASIA motor score				
UEMS	-0.113		.073*		UEMS	-0.112		.163*	
LEMS	0.018		.781*		LEMS	0.110		.172*	
K-SCIM-III	0.062		.325*		K-SCIM-III	0.175		.026*	
Sex	0.264		<.001†		Sex	0.203		.009†	
Alcohol consumption	0.046		.463†		Alcohol consumption	−0.031		.698†	
Smoking history	0.094		.136†		Smoking history	0.037		.639†	
Etiology of injury	0.160		.012†		Etiology of injury	0.035		.664†	
Extent of injury	0.013		.837†		Extent of injury	−0.096		.224†	
Complete/incomplete	0.098		.127†		Complete/incomplete	0.072		.371†	

Variables	B	β	t	*P*	Variables	B	β	t	*P*
(Constant)	−2.70		−5.32	<.001	(Constant)	−3.20		−5.50	<.001
Age (years)	−0.01	−0.21	−3.42	**.001**	Age (years)	−0.02	−3.39	−3.39	**.001**
BMI (kg/m^2^)	0.07	0.22	3.61	**<.001**	BMI (kg/m^2^)	0.08	3.12	3.12	**.002**
Sex (1 = Men)	0.45	0.19	3.07	**.002**	Sex (1 = Men)	0.43	2.35	2.35	**.020**
Etiology of injury (1 = Traumatic)	0.26	0.11	1.72	.087	K-SCIM-III	0.01	2.53	2.53	**.012**

Bold indicates statistical significance with a *P* value <.05.

ASIA = American Spinal Injury Association, BMI = body mass index, K-SCIM- III = Korean Spinal Cord Independence Measure III, LEMS = lower extremity motor, UEMS = upper extremity motor score.

* Pearson *r* correlation.

† Point-biserial correlation.

## 4. Discussion

This study analyzed BMD changes and relevant risk factors in hundreds of individuals with SCI using repeated DXA results. The distribution of demographic and SCI-related factors was similar to that in previous studies.^[30–32]^ Significant time-related changes in BMD and T-score were observed in all tested areas (lumbar spine, femoral neck, and total hip). The lumbar spine exhibited increasing trends for both BMD and T-scores, whereas the femoral neck and total hip regions showed a significant decrease in both parameters over time. These findings corroborate the results of previous studies.^[11,12,33,34]^

We divided the repeated DXA results into 1-year intervals based on the postinjury duration to identify the specific timeframe when these changes occur. In the lumbar spine, BMD and T-score values increased significantly in the “>5 years” postinjury group. Charmetant et al^[35]^ and Maïmoun et al^[36]^ reported that the BMD at the lumbar spine is preserved and, consequently, it is at a lower risk of fragility fractures, likely due to weight support in the sitting position. Conversely, substantial annual bone loss was observed in the total hip region up to 3 years postinjury and in the femoral neck region up to 2 years postinjury. Specifically, both BMD and T-score values showed the greatest decline between the “<1 year” and the “1 to 2 years” postinjury groups in both femoral neck and total hip regions. Previous studies have primarily focused on abrupt bone loss during the acute phase (mainly within the first year postinjury).^[6,12,37]^ However, this study demonstrated that significant annual bone loss occurs for up to 3 years postinjury in the femoral neck and total hip regions.

In the general population, high-risk groups susceptible to osteoporosis are identified on the basis of risk factors and receive intensive management.^[22]^ However, managing osteoporosis risk factors in individuals with SCI presents difficulties because most individuals with SCI are young or middle-aged men, and the pathophysiological mechanisms of developing osteoporosis differ from those in the general population.^[30–32,38]^ This study revealed the relevant risk factors associated with bone loss in individuals with SCI by dividing them into acute and chronic phase groups. In the acute phase, significant bone loss was more prevalent among older individuals, those with a lower BMI, and women. In contrast, in the chronic phase, in addition to age, BMI, and sex, K-SCIM-III scores showed a significant association, indicating that lower K-SCIM-III scores correlated with a significant decrease in BMD. This finding implies that regardless of the postinjury duration, older age, lower BMI, and women are risk factors for bone loss, which is consistent with well-known risk factors for osteoporosis in the general population.^[22,39]^ In the chronic phase, lower K-SCIM-III scores emerged as SCI-related risk factors for bone loss. Contrary to expectations, clinical factors related to SCI (ASIA motor score, etiology of injury, extent of injury, and AIS completeness) were not directly associated with bone loss. Our findings align with those of previous studies showing that the extent of demineralization is independent of the injury level.^[37,40]^ Additionally, it is plausible that the higher risk of fragility fractures in paraplegic individuals is not attributed to bone loss or the degree of paralysis, but rather to a higher likelihood of physical activity.^[9,41]^ Therefore, regardless of the injury duration, it is important to carefully monitor BMD in individuals who are elderly, female, or have low BMI. Additionally, updating the SCIM score may warrant closer observation during the chronic phase.

This study has several limitations. First, as this study was conducted at a single center in Korea, the findings may reflect the geographical, demographic, and medical system characteristics specific to that location. Careful consideration is required when attempting to generalize these results to other populations or countries. Second, while distal femoral and proximal tibia sites are recommended for measuring BMD in the SCI population,^[24,42]^ the present study only included the hip and lumbar spine sites. This approach offers the advantage of evaluating the SCI population using a well-established methodology. However, further research is needed to elucidate the causal relationship between observed bone loss in the hip region and the risk of fragility fractures in the lower extremities, where fractures are more prevalent. Lastly, although the correlation between bone loss and the K-SCIM-III score during the chronic phase is a new discovery, the relationship between the K-SCIM-III score and bone loss remains unclear. Establishing a cutoff score for the K-SCIM-III to categorize high-risk groups is imperative for clinical implementation.

## 5. Conclusion

This study revealed significant annual bone loss in the hip region up to 3 years postinjury. Risk factors for osteoporosis, which are important in the general population, such as old age, women, and low BMI, have also been applied to the SCI population. Additionally, an association between the K-SCIM-III score and bone loss in the chronic phase was identified. Overall, it is essential to consider the possibility of dynamic bone loss in the hip region within the initial 3 years postinjury. Incorporating the K-SCIM-III score, which assesses a patient’s independence, may provide further insight into osteoporosis risk assessment, especially during the chronic phase.

## Acknowledgments

This study was funded by a grant (NRCRSP-21TB03, NRCRSP-22TB04) by the Korea National Rehabilitation Center.

## Author contributions

**Conceptualization:** Onyoo Kim.

**Data curation:** Chaeun Mun, Keunyoung Sho.

**Formal analysis:** Chaeun Mun.

**Funding acquisition:** Onyoo Kim.

**Investigation:** Chaeun Mun, Keunyoung Sho.

**Methodology:** Chaeun Mun, Onyoo Kim, Keunyoung Sho.

**Project administration:** Chaeun Mun, Onyoo Kim.

**Resources:** Onyoo Kim.

**Software:** Keunyoung Sho.

**Supervision:** Onyoo Kim.

**Validation:** Onyoo Kim.

**Visualization:** Chaeun Mun, Keunyoung Sho.

**Writing – original draft:** Chaeun Mun.

**Writing – review & editing:** Onyoo Kim, Keunyoung Sho.

## References

[1] Szollar SM Martin EM Sartoris DJ Parthemore JG Deftos LJ. Bone mineral density and indexes of bone metabolism in spinal cord injury. Am J Phys Med Rehabil. 1998;77:28–35. 9482376 10.1097/00002060-199801000-00005

[2] Jiang SD Jiang LS Dai LY. Mechanisms of osteoporosis in spinal cord injury. Clin Endocrinol (Oxf). 2006;65:555–65. 17054455 10.1111/j.1365-2265.2006.02683.x

[3] Roberts D Lee W Cuneo RC. Longitudinal study of bone turnover after acute spinal cord injury. J Clin Endocrinol Metab. 1998;83:415–22. 9467550 10.1210/jcem.83.2.4581

[4] Battaglino RA Lazzari AA Garshick E Morse LR. Spinal cord injury-induced osteoporosis: pathogenesis and emerging therapies. Curr Osteoporos Rep. 2012;10:278–85. 22983921 10.1007/s11914-012-0117-0 PMC3508135

[5] Antoniou G Benetos IS Vlamis J Pneumaticos SG. Bone mineral density post a spinal cord injury: a review of the current literature guidelines. Cureus. 2022;14:e23434. 35494917 10.7759/cureus.23434 PMC9038209

[6] Goenka S Sethi S Pandey N Joshi M Jindal R. Effect of early treatment with zoledronic acid on prevention of bone loss in patients with acute spinal cord injury: a randomized controlled trial. Spinal Cord. 2018;56:1207–11. 30258212 10.1038/s41393-018-0195-7

[7] Abderhalden L Weaver FM Bethel M. Dual-energy X-ray absorptiometry and fracture prediction in patients with spinal cord injuries and disorders. Osteoporos Int. 2017;28:925–34. 27924381 10.1007/s00198-016-3841-y

[8] Zehnder Y Lüthi M Michel D. Long-term changes in bone metabolism, bone mineral density, quantitative ultrasound parameters, and fracture incidence after spinal cord injury: a cross-sectional observational study in 100 paraplegic men. Osteoporosis Int. 2004;15:180–9. 10.1007/s00198-003-1529-6 14722626

[9] Morse LR Battaglino RA Stolzmann KL. Osteoporotic fractures and hospitalization risk in chronic spinal cord injury. Osteoporos Int. 2009;20:385–92. 18581033 10.1007/s00198-008-0671-6 PMC2640446

[10] Dudley-Javoroski S Shields RK. Regional cortical and trabecular bone loss after spinal cord injury. J Rehabil Res Dev. 2012;49:1365–76. 23408218 10.1682/jrrd.2011.12.0245 PMC3647247

[11] Mahitthiharn K Kovindha A Kaewchur T Morse LR Pattanakuhar S. Prevalence and influencing factors of spinal cord injury-related osteoporosis and fragility fractures in Thai people with chronic spinal cord injury: a cross-sectional, observational study. J Spinal Cord Med. 2023;46:458–65. 35377283 10.1080/10790268.2022.2054763 PMC10116914

[12] Garland DE Stewart CA Adkins RH. Osteoporosis after spinal cord injury. J Orthop Res. 1992;10:371–8. 1569500 10.1002/jor.1100100309

[13] Eser P Schiessl H Willnecker J. Bone loss and steady state after spinal cord injury: a cross-sectional study using pQCT. J Musculoskelet Neuronal Interact. 2004;4:197–8. 15615125

[14] Broholm B Pødenphant J Biering-Sørensen F. The course of bone mineral density and biochemical markers of bone turnover in early postmenopausal spinal cord-lesioned females. Spinal Cord. 2005;43:674–7. 15968297 10.1038/sj.sc.3101788

[15] Varghese SM Chandy B Thomas R Tharion G. Effect of zoledronic acid on osteoporosis after chronic spinal cord injury: a randomized controlled trial. Crit Rev Phys Rehabil Med. 2016;28:85–93.

[16] Eser P Frotzler A Zehnder Y Denoth J. Fracture threshold in the femur and tibia of people with spinal cord injury as determined by peripheral quantitative computed tomography. Arch Phys Med Rehabil. 2005;86:498–504. 15759235 10.1016/j.apmr.2004.09.006

[17] Morse LR Sudhakar S Lazzari AA. Sclerostin: a candidate biomarker of SCI-induced osteoporosis. Osteoporos Int. 2013;24:961–8. 22801952 10.1007/s00198-012-2072-0 PMC3611240

[18] Javidan AN Sabour H Latifi S. Evaluation of bone mineral loss in patients with chronic traumatic spinal cord injury in Iran. J Spinal Cord Med. 2014;37:744–50. 24621041 10.1179/2045772313Y.0000000192 PMC4231962

[19] Garland DE Adkins RH Stewart CA Ashford R Vigil D. Regional osteoporosis in women who have a complete spinal cord injury. J Bone Joint Surg Am. 2001;83:1195–200. 11507128 10.2106/00004623-200108000-00009

[20] Kiratli BJ Smith AE Nauenberg T Kallfelz CF Perkash I. Bone mineral and geometric changes through the femur with immobilization due to spinal cord injury. J Rehabil Res Dev. 2000;37:225–33. 10850829

[21] Wilson KE. Bedford: Hologic Inc; 2011. Practical considerations when replacing a DXA system. https://hologiced.com/wp-content/uploads/2018/06/Wilson-KE.-Practical-Considerations-When-Replacing-a-DXA-System.pdf. Accessed September 4, 2024.

[22] Prevention and management of osteoporosis: Report of a WHO scientific group. World Health Organization. World Health Organization; 1970. https://iris.who.int/handle/10665/42841.

[23] Hanson J. Standardization of femur BMD. J Bone Miner Res. 1997;12:1316–7. 9258764 10.1359/jbmr.1997.12.8.1316

[24] Morse LR Biering-Soerensen F Carbone LD. Bone mineral density testing in spinal cord injury: 2019 ISCD Official Position. J Clin Densitom. 2019;22:554–66. 31501005 10.1016/j.jocd.2019.07.012

[25] Bogenschutz MP Ross S Bhatt S. Percentage of heavy drinking days following psilocybin-assisted psychotherapy vs placebo in the treatment of adult patients with alcohol use disorder. a randomized clinical trial. JAMA Psychiatry. 2022;79:953–62. 36001306 10.1001/jamapsychiatry.2022.2096 PMC9403854

[26] Centers for Disease Control and Prevention. Alcohol and public health – What is excessive drinking?. 2024. https://www.cdc.gov/drinklessbeyourbest/excessivedrinking.html.

[27] International standards for neurological classification of sci (ISNCSCI) worksheet. American Spinal Injury Association. 2024. https://asia-spinalinjury.org/international-standards-neurological-classification-sci-isncsci-worksheet/.

[28] Itzkovich M Gelernter I Biering-Sorensen F. The Spinal Cord Independence Measure (SCIM) version III: reliability and validity in a multi-center international study. Disabil Rehabil. 2007;29:1926–33. 17852230 10.1080/09638280601046302

[29] Cho DY Shin H-I Kim H-R. Reliability and validity of the Korean version of the spinal cord independence measure III. Am J Phys Med Rehabil. 2020;99:305–9. 31651451 10.1097/PHM.0000000000001327

[30] Kang Y Ding H Zhou H. Epidemiology of worldwide spinal cord injury: a literature review. J Neurorestoratology. 2018;Volume 6:1–9.

[31] Ning G-Z Wu Q Li Y-L Feng S-Q. Epidemiology of traumatic spinal cord injury in Asia: a systematic review. J Spinal Cord Med. 2012;35:229–39. 22925749 10.1179/2045772312Y.0000000021 PMC3425879

[32] National Spinal Cord Injury Statistical Center. The 2019 annual statistical report for the spinal cord injury model systems. Birmingham, Alabama: National Spinal Cord Injury Statistical Center; 2021;25–36.

[33] Jones L Legge M Goulding A. Intensive exercise may preserve bone mass of the upper limbs in spinal cord injured males but does not retard demineralisation of the lower body. Spinal Cord. 2002;40:230–5. 11987005 10.1038/sj.sc.3101286

[34] Bauman WA Kirshblum S Cirnigliaro C Forrest GF Spungen AM. Underestimation of bone loss of the spine with posterior-anterior dual-energy X-ray absorptiometry in patients with spinal cord injury. J Spinal Cord Med. 2010;33:214–20. 20737794 10.1080/10790268.2010.11689698 PMC2920114

[35] Charmetant C Phaner V Condemine A Calmels P. Diagnosis and treatment of osteoporosis in spinal cord injury patients: a literature review. Ann Phys Rehabil Med. 2010;53:655–68. 21094110 10.1016/j.rehab.2010.10.001

[36] Maïmoun L Ben Bouallègue F Gelis A. Periostin and sclerostin levels in individuals with spinal cord injury and their relationship with bone mass, bone turnover, fracture and osteoporosis status. Bone. 2019;127:612–9. 31351195 10.1016/j.bone.2019.07.019

[37] Frey-Rindova P De Bruin E Stüssi E Dambacher MA Dietz V. Bone mineral density in upper and lower extremities during 12 months after spinal cord injury measured by peripheral quantitative computed tomography. Spinal Cord. 2000;38:26–32. 10762194 10.1038/sj.sc.3100905

[38] Cervinka T Lynch C Giangregorio L. Agreement between fragility fracture risk assessment algorithms as applied to adults with chronic spinal cord injury. Spinal Cord. 2017;55:985–93. 28607522 10.1038/sc.2017.65

[39] van der Voort D Geusens PP Dinant G. Risk factors for osteoporosis related to their outcome: fractures. Osteoporos Int. 2001;12:630–8. 11580076 10.1007/s001980170062

[40] Dauty M Perrouin Verbe B Maugars Y Dubois C Mathe JF. Supralesional and sublesional bone mineral density in spinal cord-injured patients. Bone. 2000;27:305–9. 10913927 10.1016/s8756-3282(00)00326-4

[41] Bauman WA Cardozo CP. Osteoporosis in individuals with spinal cord injury. PM R. 2015;7:188–201; quiz 201. 25171878 10.1016/j.pmrj.2014.08.948

[42] Wright NC Looker AC Saag KG. The recent prevalence of osteoporosis and low bone mass in the United States based on bone mineral density at the femoral neck or lumbar spine. J Bone Miner Res. 2014;29:2520–6. 24771492 10.1002/jbmr.2269 PMC4757905

